# Modulation of *Rxr*α Expression in Mononuclear Phagocytes Impacts on Cardiac Remodeling after Ischemia-Reperfusion Injury

**DOI:** 10.3390/biomedicines10061274

**Published:** 2022-05-30

**Authors:** Saskia Räuber, Maximilian Fischer, Denise Messerer, Vanessa Wimmler, Kumaraswami Konda, Andrei Todica, Michael Lorenz, Anna Titova, Christian Schulz, Tobias Weinberger

**Affiliations:** 1 Medical Clinic I., Department of Cardiology, University Hospital, Ludwig Maximilian University, 81377 Munich, Germany; saskiajanina.raeuber@med.uni-duesseldorf.de (S.R.); maximilian.fischer@med.uni-muenchen.de (M.F.); denise.messerer@med.uni-muenchen.de (D.M.); vanessa.wimmler@gmx.de (V.W.); Kumaraswami.Konda@med.uni-muenchen.de (K.K.); michael.lorenz@med.uni-muenchen.de (M.L.); anna.titova@med.uni-muenchen.de (A.T.); 2 Department of Neurology, Medical Faculty, Heinrich Heine University of Düsseldorf, 40225 Düsseldorf, Germany; 3 Department of Nuclear Medicine, Ludwig Maximilian University, 81377 Munich, Germany; andrei.todica@med.uni-muenchen.de; 4 DZHK (German Centre for Cardiovascular Research), Partner Site Munich Heart Alliance, 80802 Munich, Germany

**Keywords:** macrophages, mononuclear phagocytes, ischemia/reperfusion injury, retinoid receptors, inflammation

## Abstract

Retinoid X receptors (RXRs), as members of the steroid/thyroid hormone superfamily of nuclear receptors, are crucial regulators of immune response during health and disease. RXR subtype expression is dependent on tissue and cell type, RXRα being the relevant isoform in monocytes and macrophages. Previous studies have assessed different functions of RXRs and positive implications of RXR agonists on outcomes after ischemic injuries have been described. However, the impact of a reduced *Rxrα* expression in mononuclear phagocytes on cardiac remodeling after myocardial infarction (MI) has not been investigated to date. Here, we use a temporally controlled deletion of *Rxrα* in monocytes and macrophages to determine its role in ischemia-reperfusion injury. We show that reduced expression of *Rxrα* in mononuclear phagocytes leads to a decreased phagocytic activity and an accumulation of apoptotic cells in the myocardium, reduces angiogenesis and cardiac macrophage proliferation in the infarct border zone/infarct area, and has an impact on monocyte/macrophage subset composition. These changes are associated with a greater myocardial defect 30 days after ischemia/reperfusion injury. Overall, the reduction of *Rxrα* levels in monocytes and macrophages negatively impacts cardiac remodeling after myocardial infarction. Thus, RXRα might represent a therapeutic target to regulate the immune response after MI in order to improve cardiac remodeling.

## 1. Introduction

Myocardial infarction (MI) is a common and potentially lethal condition caused by the disruption of coronary artery blood flow leading to myocardial ischemia. Technical and conceptual advances over the last decades have facilitated early diagnosis and therapy of MI; however, a considerable number of patients still develop heart failure after an ischemic event [1]. Thus, new therapeutic approaches are needed to improve cardiac remodeling and outcome after MI. Strict regulation of the immune response is crucial to maintain cardiac function after MI as impaired resolution of inflammation leads to left-ventricular dilatation and has a negative impact on the outcome after an ischemic injury [2,3,4]. Macrophages, as a part of the innate immune system, are the most abundant immune cells in the heart. They mediate cardiac homeostasis and are key players in the resolution of inflammation after ischemic injury of the heart [2]. They have the ability to proliferate and self-renew and are able to exist mostly independently from blood-monocytes in the absence of cardiac injury [5]. Depletion of cardiac macrophages increases the left-ventricular diameter, promotes wall-thinning, and leads to a higher mortality rate [6]. Cardiac tissue macrophages abundantly express the Fractalkine receptor CX3CR1. We here used Tamoxifen-inducible *Cx3cr1-Cre* mice to modulate *Rxrα* expression in mononuclear phagocytes.

Retinoid X Receptors (RXRs) are part of the steroid/thyroid hormone superfamily of nuclear receptors operating as transcription factors. The three subtypes (RXRα, RXRβ, and RXRγ) are differently expressed in cells and tissues [7,8], with RXRα being the predominant subtype in monocytes and macrophages [8,9]. In the course of acute inflammation, RXRs are involved in a variety of regulatory processes (e.g., chemokine expression, cell migration, cell survival, and angiogenesis) [8,9,10,11,12,13,14]. RXRs possess anti-inflammatory capacities and represent important mediators during the resolution of inflammation. Activation of RXRs reduces the expression of inflammatory chemokines as well as degradation of the extracellular matrix [15,16] while upregulating the release of anti-inflammatory molecules [12,13] and inducing angiogenesis [14]. The importance of RXRs in the context of immune regulation could be demonstrated using mice with a myeloid-specific deletion of RXRα. Respective animals show a reduced self-tolerance and develop autoimmune kidney disease. Impaired phagocytic activity of macrophages could be identified as a main contributor to immune dysregulation. A reduced uptake of cell debris by macrophages leads to an increased number of apoptotic cells in the kidney and disrupts the formation of anti-inflammatory macrophages [17]. In mice subjected to strokes, RXRα-deletion in myeloid cells reduced functional recovery and increased brain atrophy compared to the control mice [18]. Different studies on the pathophysiological role of RXRs have been performed so far [10,11,14,17,19,20]; however, the function of RXRα in mononuclear phagocytes after MI remains elusive to date.

In this study we aimed to decipher the role of RXRα in mononuclear phagocytes on cardiac remodeling after MI using a cell-specific conditional deletion model. We provide evidence for the involvement of RXRα in monocytes and macrophages in cardiac remodeling after MI. Our results indicate that a reduced expression of *Rxrα* is associated with an increase in infarct size during the later stages of MI. Mechanistically, we identified a reduced mononuclear phagocytic activity paralleled by higher amounts of apoptotic cells in the myocardium, a lower vessel density in the infarct border zone, an impaired proliferation of cardiac macrophages, and alterations in monocyte and macrophage populations in the blood and myocardium in mice with a reduced *Rxrα* expression in mononuclear phagocytes. Together, RXRα modulates the cardiac immune response and impacts the outcome after ischemia-perfusion injury.

## 2. Materials and Methods

### 2.1. Mice

**Figure 1:**
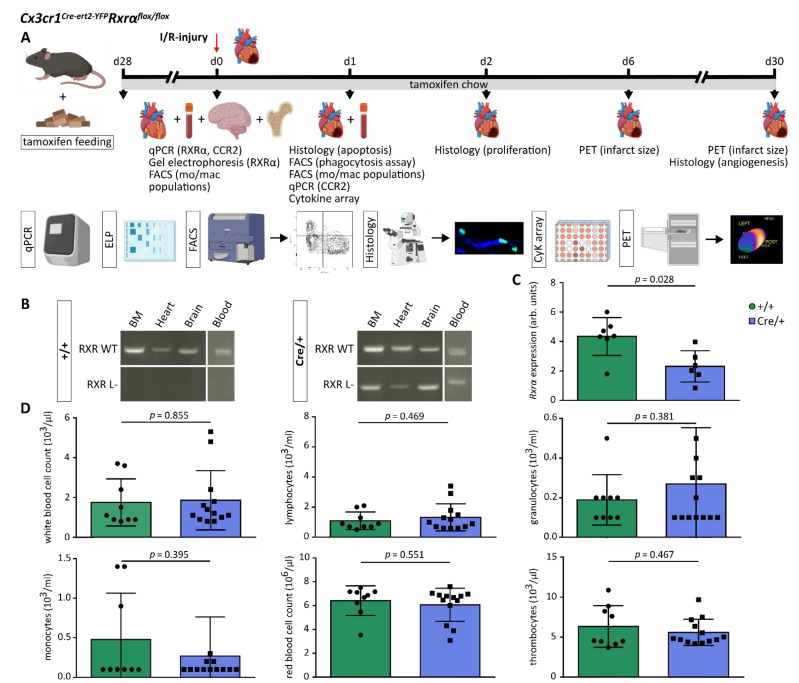
*Cx3cr1^Cre-ert2-YFP^Rxrα^flox/flox^* mice show reduced expression of *Rxrα* in blood monocytes, which has no impact on PB parameters. (**A**) Study design (created with BioRender.com); (**B**) *Rxrα* wildtype allele and *Rxrα* L- allele expression in PB and different tissues of *Cx3cr1ΔRxrα* and control mice assessed by PCR and gel electrophoresis; (**C**) comparison of blood monocyte *Rxrα* expression between *Cx3cr1ΔRxrα* and control mice; (**D**) analysis of different blood parameters in *Cx3cr1ΔRxrα* and control mice. Arb: arbitrary; BM: bone marrow; CCR2: C-C chemokine receptor type 2; control (+/+): control mice [littermates with a floxed Rxrα allele but without Cre expression]; Cx3cr1ΔRxrα (Cre/+): mice with a floxed Rxrα allele and Cre expression; CyK: cytokine; ELP: electrophoresis; FACS: Fluorescence activated cell sorting; I/R injury: Ischemia/Reperfusion injury; mac: macrophage; mo: monocyte; PB: peripheral blood; PET: positron emission tomography; Rxr: Retinoid X receptor; qPCR: real-time polymerase chain reaction.

*Rxrα*^flox/flox^ mice have been previously described [21]. In order to achieve a selective reduction of *Rxrα* expression in mononuclear phagocytes, *Rxrα*^flox/flox^ mice were crossed with *Cx3cr1^Cre-ert2^* mice. Tamoxifen was administered orally at a dose of 40 mg/kg per day to induce Cre activity as described before [21,22]. Feeding was started 28 days prior to the left anterior descending artery (LAD) ligation (Figure 1A). Mice with a floxed *Rxrα* allele and Cre expression are referred to as *Cx3cr1ΔRxrα*. Littermates with a floxed *Rxrα* allele but without Cre expression were used as controls (control). All mice were kept in a specific pathogen-free environment. Animals were 12–16 weeks of age at the time of the experiments. Animal studies were performed in accordance with the respective ethical regulations and were approved by the local regulatory agency (Regierung von Oberbayern, Munich, Germany, record numbers 55.2-1-54-2532-76-13, date of approval 21/05/2014, and ROB-55.2-2532.Vet_02-19-17; date of approval 01/07/2019).

### 2.2. Ischemia/Reperfusion (I/R) Injury

I/R injury was performed by transient ligation of the LAD. Mice were anesthetized using isoflurane 5.0% p.i., midazolam i.p. at a dose of 5.0 mg/kg, and medetomidine i.p. at a dose of 0.5 mg/kg. Intraoperative analgesia was obtained using fentanyl i.p. at a dose of 0.05 mg/kg, and buprenorphine was given i.p. at a dose of 0.1 mg/kg postoperatively twice a day for 5 days. An incision was made at the level of the second intercostal space and the LAD visualized. Next, the LAD was ligated using a polypropylene suture (Prolene 8-0, Ethicon, Johnson&Johnson Medical, Norderstedt, Germany) and a space holder. After a period of 60 min, reperfusion was achieved by the removal of the space holder as well as the ligature.

### 2.3. Tissue and Organ Harvesting

Organs were harvested at different time points, depending on the experimental setup (Figure 1A). Mice were anesthetized as described above, and an incision of the chest was made at the level of the second intercostal space. Blood was collected by cardiac puncturing. Heparin was used to avoid blood clotting. Mice were euthanized by cervical dislocation. The heart was dissected and perfused with sodium chloride. The skull was cut open alongside the sutures and the brain was extracted. For bone marrow (BM) analysis, the long bones were dissected and removed.

### 2.4. Blood Analysis

Analysis of blood samples was performed using the ABX Micros 60 (Horiba, Kyoto, Japan). 100 µL blood were transferred to a capillary blood collection system (Microvette, Sarstedt Ag& Co, Nümbecht, Germany) and analysis was performed according to the manufacturer′s instructions.

### 2.5. FACS (Sorting)

#### 2.5.1. Preparation of Blood and BM Samples

The endings of long bones were removed on both sides, and the bones were perfused with Phosphate-Buffered Saline 1× (PBS) and 2% Fetal Bovine Serum (FBS) to harvest cells. Centrifugation of BM/blood cells was performed for 5 min at 350× *g* and 4 °C. Cells were suspended in red-blood-cell-lysis-buffer (20 g ammonium chloride 1.5 M, 2.5 g potassium bicarbonate 0.1 M, 5 mL EDTA 0.5 M, and 250 mL distilled water). For blood samples, centrifugation and lysis were repeated. Samples were transferred to a 96-Well plate, Purified Rat Anti-Mouse CD16/CD32 (BDBioscience, Franklin Lakes, NJ, USA, ref. no. 553141) and staining antibodies (Table S1) were added. After an incubation period of 20 min, samples were centrifuged and diluted in FACS buffer. Samples were analyzed using a Cell Analyzer BD LSR Fortessa (Franklin Lakes, NJ, USA). ‘FlowJo’ (version 10.2, Becton, Dickinson & Company, Ashland, OR, USA) was used for data analysis. Isolation of cell populations for further analysis was performed using a Beckman Coulter Astrios (Beckman Coulter, Brea, OR, USA).

#### 2.5.2. Preparation of Heart and Brain Samples

Tissue was cut into small pieces and added to a solution allowing enzymatic tissue digestion (1 mL Collagenase XI, 240 µL DNase I, 1.8 mL Collagenase I, 120 µL Hyaluronidase and 2.8 mL PBS). An Eppendorf ThermoMixer^®^ (Eppendorf AG, Hamburg, Germany) was used for incubation (30 min) at 37 °C and 400 rpm.

Digested tissues were mechanically dissociated using a 70 µm strainer and a syringe plunger. After centrifugation of the samples, the cells were transferred to a 96-Well-plate. Purified Rat Anti-Mouse CD16/CD32 and CD45 MicroBeads (Miltenyi Biotec, Bergisch-Gladbach, Germany, ref. no. 130-052-301) were added and incubated for 15 min at 4 °C. The cells were washed, staining antibodies (Table S1) were applied, and incubation took place for 30 min at 4 °C. Magnetic cell separation was performed as recommended in the user manual.

#### 2.5.3. Phagocytosis Assay Using pHrodo™ Red BioParticles^®^

PHrodo™ Red BioParticles^®^ (ThermoFisher, Waltham, MA, USA, ref. no. P35361) were used to analyze the phagocytic activity of blood monocytes. Blood samples were prepared as described above. The following steps were performed according to the manufacturer′s instructions.

Briefly, blood samples were prepared as described in Section 2.5.1. Following the last centrifugation step, the cells were diluted in DMEM/F-12 (HEPES, no phenol red) and transferred to a 96-well plate (flat bottom). Cytochalasin D was added to one well serving as the negative control. The cells were incubated for 1 h at 37 °C. PHrodo™ Red BioParticles were thawed and diluted in Live Cell Imaging Solution. After 1 h, cell culture medium was removed and 100 µL pHrodo™ Red BioParticles were added per well. Incubation was repeated for 1 h at 37 °C. Next, pHrodo™ Red BioParticles were removed and 100 µL detachment buffer was added per well (500 mL PBS, 0.09 g glucose, 3 mL EDTA). Incubation was performed for 15 min at 37 °C. Subsequently, cells were centrifuged for 5 min at 350× *g* and 4 °C. Supernatant was discarded and cells were diluted in FACS buffer.

Cell Analyzer BD LSR Fortessa was used to analyze the samples.

### 2.6. Immunofluorescence Microscopy (IF)

Mice were euthanized by cervical dislocation under terminal anesthesia. Intracardiac injection of 20 mL 4% PFA was performed. The heart was removed, washed with PBS, and transferred into 30% sucrose. After an overnight incubation at 4 °C, the heart was cut into three pieces and embedded in Tissue-Tek OCT (Sakura Finetek, Alphen aan den Rijn, The Netherlands). Cryoblocks were sliced into 12-μm sections using a cryotome. Multiple slices from different areas of the heart were cut. Slices were fixed with 4% PFA, blocked, and permeabilized with 0.5% Saponin, 10% goat serum, and 1% BSA (Albumin fraction V). After 1 h, the excessive fluid was removed and 50 µL of diluted antibodies (Table S1) were added per section. The incubation took place for 1 h at room temperature. Afterwards, the slides were washed three times in PBS, diluted corresponding secondary antibodies (Table S1) were added, and incubation was performed for 1 h at room temperature.

Washing was repeated and staining with a Wheat Germ Agglutinin Alexa Fluor™ 350 conjugated antibody (Table S1) was performed to locate the infarction area on d30 after I/R-injury. The border zone was defined as the area within 500 µm around the infarct zone. Washing was repeated and the slices were covered with Fluorescence Mounting Medium (DAKO GmbH, Jena, Germany). Samples were analyzed with a Zeiss Axio Imager M2 equipped with an AxioCam MRm (Zeiss, Oberkochen, Germany). Picture analysis was performed with the software ‘AxioVision SE64 Rel. 4.9.1′ (Zeiss, Oberkochen, Germany).

### 2.7. Quantification of Apoptotic Cells in the Heart on d1 after MI

The number of apoptotic cells in the infarcted myocardium was quantified using the ApopTag Plus Fluorescein In Situ Apoptosis Detection Kit (Merck, Burlington, VT, USA, ref. no. S7110) according to the manufacturer′s instructions.

### 2.8. (Real Time Quantitative) PCR

Cell populations were isolated using a Beckman Coulter Astrios. RNA isolation, and reverse transcription was performed with a RNeasy^®^ Plus Micro Kit (Qiagen, Hilden, Germany ref. no. 74034) and a High-Capacity cDNA Reverse Transcription Kit (ThermoFisher, Waltham, MA, USA, ref. no. 4368813). For qPCR, SsoAdvanced™ Universal SYBR^®^ Green Supermix (BIO-RAD, Hercules, CA, USA, ref. no. 1725270) was used.

The following primers were applied: primer ZO243 (binding site: exon 3) and ZO244 (binding site: exon 4) (Eurofins Scientific, Luxemburg) to evaluate the expression of the Rxrα wildtype allele; primer ZO243 and UD196 (binding site: in between exon 4 and 5) (Eurofins Scientific, Luxemburg) for evaluation of the Rxrα L- allele expression; and Mm_Ccr2_3_SG QuantiTect Primer Assay (Qiagen, Hilden, Germany, ref. no. QT02522849) to analyze CCR2 expression. Primers for the quantification of Rxrα expression were generated based on the gene sequence of the Rxrα gene and were provided by Eurofins Genomics (sequence of upward-primer: CGCTCCTCAGGCAAACACTATG, sequence of downward-primer: GGTTCCGCTGTCTCTTGTCG). GAPDH was used as housekeeping gene: Mm_Gapdh_3_SG QuantiTect Primer Assay (Qiagen, Hilden, Germany, ref. no. QT01658692).

### 2.9. Gel Electrophoresis

Agarose gel was made using 3 g agarose and 120 mL TBE buffer (2.5% gel). Ingredients were mixed and microwaved for 2.5 min. Six microliters of Roti^®^-GelStain (Carl Roth, Karlsruhe, Germany, ref. no. 3865.1) was added and the gel was poured into a gel chamber. Twenty microliters of each cDNA sample was mixed with 4 µL DNA Gel Loading Dye 6× (ThermoFisher, Waltham, MA, USA, ref. no. R0611) and briefly centrifuged at 2000 rpm. Samples and GeneRuler 100 bp DNA Ladder (ThermoFisher, Waltham, MA, USA, ref. no. SM0241) were added to the agarose gel and electrophoresis was started. Gel Doc 2000 (BIO-RAD, Hercules, CA, USA) was used for imaging.

### 2.10. Cytokine Array

Analysis of cardiac cytokine and chemokine expression was performed using the Proteome ProfilerTM Mouse XL Cytokine Array Kit (R&D Systems, ref. no. ARY028). Hearts were cut into three pieces. Apexes were used to make tissue lysates. One milliliter of the RIPA Lysis and Extraction Buffer (ThermoFisher, Waltham, MA, USA, ref. no. 89901), one hundred microliters of EDTA, and one hundred microliters of the Halt™ Protease Inhibitor Cocktail (100×) (ThermoFisher, Waltham, MA, USA, ref. no. 78430) were mixed and added to the tissue. Samples were homogenized with an Ultra-Turrax, centrifuged at 10,000× *g* and 4 °C for 5 min, and protein concentration was measured using the Pierce™ BCA Protein Assay Kit (ThermoFisher Waltham, MA, USA, ref. no. 23225) and a Tecan GENios Microplate Reader. The following steps were performed as described in the user manual of the kit. The software ‘Image Studio™ Lite’ was used for data analysis. Analytes are summarized in Table S2.

### 2.11. Positron Emission Tomography (PET) Imaging

Small-animal imaging was performed in cooperation with the Department of Nuclear Medicine of the LMU Munich. The tracer [^18^F] FDG was provided by the company PET Net GmbH. Mice were narcotized using isoflurane 2.0% p.i. and the tracer was injected in a lateral tail vein. After 20 min, acquisition was started using an Inveon P120 μPET Scanner. An emission scan was performed for 15 min, followed by a transmission scan for 7 min. The software ‘Inveon acquisition and research workplace’ was used for technical processing. Evaluation of the reconstructed images was performed using the commercially available software QPS/QGS (QUAD, Los Angeles, CA, USA), as described previously [23].

### 2.12. Statistical Analysis

Statistical analysis was performed using the software ‘GraphPad PRISM’ (version 6.2, GraphPad Software, San Diego, CA, USA) and ‘Microsoft Excel 2016′ (Microsoft, Redmond, DC, USA). The Fisher–Pitman test was used for small animal numbers (*n* < 15) and the Welch′s *t*-test for *n* > 15. *p*-value < 0.05 was considered significant. All results are shown as mean ± standard deviation.

## 3. Results

### 3.1. Rxrα Expression Is Reduced in Blood Monocytes of Cx3cr1^Cre-ert2-YFP^Rxrα^flox/flox^ Mice

RXRs are involved in the regulation of different genes, and various studies on the function of RXRs during health and disease have been previously performed [7,8]. However, the impact of a reduced expression of *Rxrα* in monocytes and macrophages on cardiac remodeling after MI has not been assessed so far. We used a tamoxifen-inducible Cre-lox-system specific for mononuclear phagocytes (*Cx3cr1^Cre-ert2^*) to delete *Rxrα* (*Rxrα^flox/flox^*) and combined it with an I/R-model to study the function of RXRα. Using this depletion model, we excluded influences of *Rxrα* depletion during developmental stages as well as deletion in other cell types.

In *Cx3cr1^Cre-ert2-YFP^Rxrα^flox/flox^* mice, deletion of exon 4 of the *Rxrα* gene (encoding parts of the DNA-binding domain) by the Cre-enzyme leads to the expression of RXRα molecules that cannot maintain their physiological function [21]. We analyzed the expression of the *Rxrα* wildtype and *Rxrα* L- allele in different tissues (BM, heart, brain, blood) after 4 weeks of tamoxifen chow (40mg/kg per day) in Cre/+ (further called *Cx3cr1ΔRxrα*) and control mice. In the control mice, by contrast, only the expression of the *Rxrα* wildtype allele could be detected, *Cx3cr1ΔRxrα* mice expressed the *Rxrα* L- allele as well as the *Rxrα* wildtype allele (Figure 1B).

Next, we quantified the expression of intact *Rxrα* (henceforth termed *Rxrα* expression for simplicity) in blood monocytes using qPCR. *Rxrα* expression in blood monocytes of *Cx3cr1ΔRxrα* mice was significantly reduced compared to the control animals (Figure 1C).

Overall, we established a mouse strain in which the *Rxrα* expression was reduced by approximately 50% in the myeloid cell population, allowing to determine the impact of reduced *Rxrα* expression on cardiac remodeling after MI.

### 3.2. Reduced Rxrα Expression in Myeloid Cells Has no Impact on Haematopoiesis

The relevance of RXRα during hematopoiesis has been a subject of debate. While some studies claim that RXRs are involved in hematopoiesis, others could not find an effect of a *Rxrα* deletion on the formation of blood cells [24,25,26].

We analyzed blood samples of *Cx3cr1ΔRxrα* and control mice on d30 after I/R-injury to evaluate the impact of a myeloid-specific reduction of *Rxrα* expression on different blood values. We could not detect significant changes in white blood cell counts, in the number of lymphocytes, granulocytes, and monocytes between *Cx3cr1ΔRxrα* and the control animals. Likewise, no differences in red blood cell counts, and thrombocytes could be found between the groups (Figure 1D).

Thus, our data suggest that a reduced expression of *Rxrα* in monocytes and macrophages has no effect on blood cell counts.

### 3.3. RXRα Influences Monocyte and Macrophage Subset Composition

Monocytes and macrophages can be characterized and divided into subsets based on expression of cell surface markers. Those subsets have different functions, possess distinct gene expression patterns, and show dynamic changes after cardiac injury [3,27,28].

**Figure 2:**
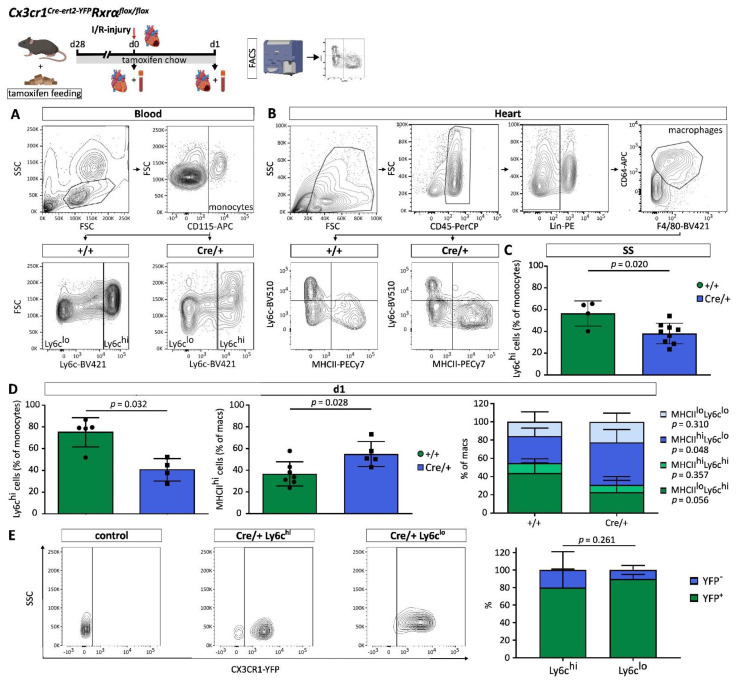
Reduced *Rxrα* expression in myeloid cells influences monocyte and macrophage subset composition. (**A**) FACS gating strategy to assess monocyte subsets in peripheral blood; (**B**) FACS gating strategy to analyze cardiac macrophage populations; (**C**) amount of Ly6c^hi^ monocytes in PB during steady state in *Cx3cr1ΔRxrα* compared to control mice; (**D**) proportions of Ly6c^hi^ monocytes in PB and MHCII^hi^ macrophages in the heart of *Cx3cr1ΔRxrα* in relation to control mice on d1 after I/R injury; (**E**) intrinsic YFP expression in Ly6c^hi^ versus Ly6c^lo^ monocytes of Cre/+ compared to control mice. Flowchart was created with BioRender.com. control (+/+): control mice [littermates with a floxed Rxrα allele but without Cre expression]; *Cx3cr1ΔRxrα* (Cre/+): mice with a floxed Rxrα allele and Cre expression; FACS: fluorescence activated cell sorting; FSC: forward scatter; hi: high; I/R injury: Ischemia/Reperfusion injury; lo: low; Lin: Lineage; macs: macrophages; Rxr: Retinoid X receptor; SS: Steady state; SSC: sideward scatter.

We studied the effect of a reduced *Rxrα* expression in myeloid cells on monocyte and macrophage subset composition applying FACS and qPCR (Figure 2A,B and Figure S1). Quantification of Ly6c expression revealed significantly lower proportions of Ly6c^hi^ blood monocytes in *Cx3cr1ΔRxrα* mice during steady state (SS) and on d1 after MI (Figure 2C,D).

In cardiac macrophages, expression of MHCII was higher in *Cx3cr1ΔRxrα* compared to the control mice on d1 after MI (Figure 2D). In-depth analysis of macrophage subsets showed that, mainly, the MHCII^hi^Ly6c^lo^ macrophage population was expanded in the heart of *Cx3cr1ΔRxrα* mice in relation to control mice (Figure 2D). No changes in CCR2 expression analyzed by qPCR in blood monocytes and cardiac macrophages could be noted between groups (Figure S1).

We further analyzed the intrinsic YFP signal of Ly6c^hi^ and Ly6c^lo^ blood monocytes as a surrogate of *Cx3cr1* and Cre expression in *Cx3cr1ΔRxrα* mice and found relevant levels of YFP in both monocyte populations compared to control animals indicating similar Cre-efficiency (Figure 2E).

Taken together, this indicates that RXRα has an impact on monocyte and macrophage subset composition during SS and acute state after MI.

### 3.4. Reduced Expression of Rxrα in Monocytes and Macrophages Increases Infarct Size after MI

**Figure 3:**
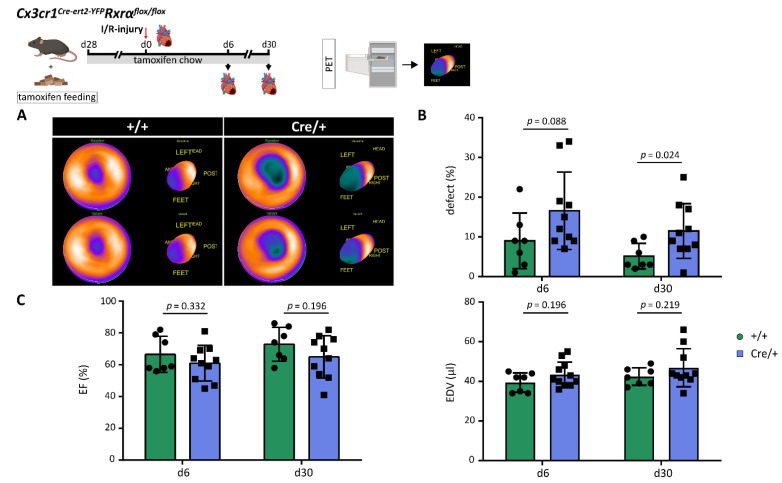
Reduction of myeloid *Rxrα* expression increases infarct size after I/R injury. (**A**) PET imaging to assess the cardiac defect on d6 and d30 after MI; (**B**) comparison of cardiac defect on d6 and d30 after MI between *Cx3cr1ΔRxrα* and control mice; (**C**) assessment of functional parameters (EDV and EF) on d6 and d30 after MI in *Cx3cr1ΔRxrα* and control mice. Flowchart was created with BioRender.com. control (+/+): control mice [littermates with a floxed Rxrα allele but without Cre expression]; *Cx3cr1ΔRxrα* (Cre/+): mice with a floxed Rxrα allele and Cre expression; EDV: end-diastolic volume; EF: ejection fraction; I/R injury: Ischemia/Reperfusion injury; PET: positron emission tomography; Rxr: Retinoid X receptor.

Previous studies indicated protective effects of RXR agonists after ischemic brain injury [18,29]. Thus, we sought to determine the impact of a reduced *Rxrα* expression on infarct size (defect) measured by the total perfusion deficit (TPD) using FDG-PET. Infarct size was assessed on d6 and d30 after MI and compared between *Cx3cr1ΔRxrα* and control mice (Figure 3A). On d30 after MI, the infarct size in *Cx3cr1ΔRxrα* animals was significantly higher in relation to the control mice (Figure 3B). Evaluation of functional parameters, end-diastolic volume (EDV), and ejection fraction (EF) did not reveal significant differences between groups (Figure 3C).

Overall, the reduction of RXRα levels in myeloid cells is associated with an increase in infarct size, as measured by the TPD after MI.

### 3.5. Phagocytic Function of Myeloid Cells Is Altered by a Reduced Rxrα-Expression

An impaired phagocytic activity of macrophages could already be identified as one crucial parameter contributing to dysregulated immune response and loss of self-tolerance after myeloid-specific RXRα deletion [17]. As we found differences in healing after MI, we analyzed the phagocytic function of myeloid cells in *Cx3cr1^Cre-ert2-YFP^Rxrα^flox/flox^* mice.

**Figure 4:**
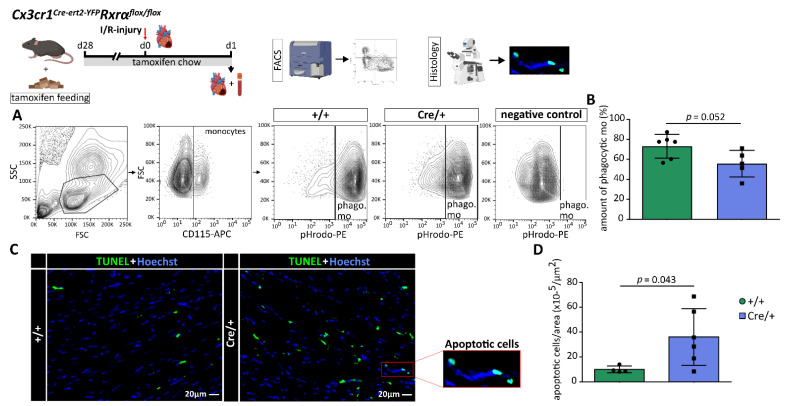
Reduction of *Rxrα* expression in myeloid cells impairs their phagocytic function and is associated with an increase in apoptotic cells after I/R injury. (**A**) FACS gating strategy to analyze the phagocytic function of PB monocytes; (**B**) comparison of the phagocytic activity of PB monocytes from *Cx3cr1ΔRxrα* and control mice; (**C**) co-staining of apoptotic cells using an ApopTag Plus Fluorescein In Situ Apoptosis Detection Kit and the nuclear counterstain Hoechst; (**D**) quantification of apoptotic cells per area in the heart of *Cx3cr1ΔRxrα* compared to control mice on d1 after MI. Flowchart was created with BioRender.com. control (+/+): control mice [littermates with a floxed Rxrα allele but without Cre expression]; *Cx3cr1ΔRxrα* (Cre/+): mice with a floxed Rxrα allele and Cre expression; FACS: fluorescence activated cell sorting; FSC: forward scatter; I/R injury: Ischemia/Reperfusion injury; mo: monocytes; phago.: phagocytic; Rxr: Retinoid X receptor; SSC: sideward scatter.

To investigate the effect of a reduced *Rxrα* expression in myeloid cells on phagocytosis, we performed a phagocytosis assay using pHrodo™ Red BioParticles^®^ (Methods, Figure 4A). Comparison of phagocytic activity of blood monocytes from *Cx3cr1ΔRxrα* showed a decrease of phagocytic activity in comparison to the control mice (Figure 4B).

Next, we were interested in whether a reduced *Rxrα* expression in myeloid cells has an impact on the number of apoptotic cells in the heart after MI. Histological analysis revealed that *Cx3cr1ΔRxrα* animals had significantly more apoptotic cells in the infarcted myocardium on d1 after ischemic injury compared to the control mice (Figure 4C,D).

Taken together, phagocytic function of blood monocytes is altered by reduced *Rxrα*-expression and paralleled by an increased number of apoptotic cells in the heart after MI.

### 3.6. Proliferation of Cardiac Macrophages Is Dependent on RXRα

Given the inhibitory effects of RXRs on clonal cell proliferation, RXR agonists are already used for the treatment of leukemia [30,31]. However, the influence of RXRα on local proliferation of cardiac macrophages has not been previously investigated.

**Figure 5:**
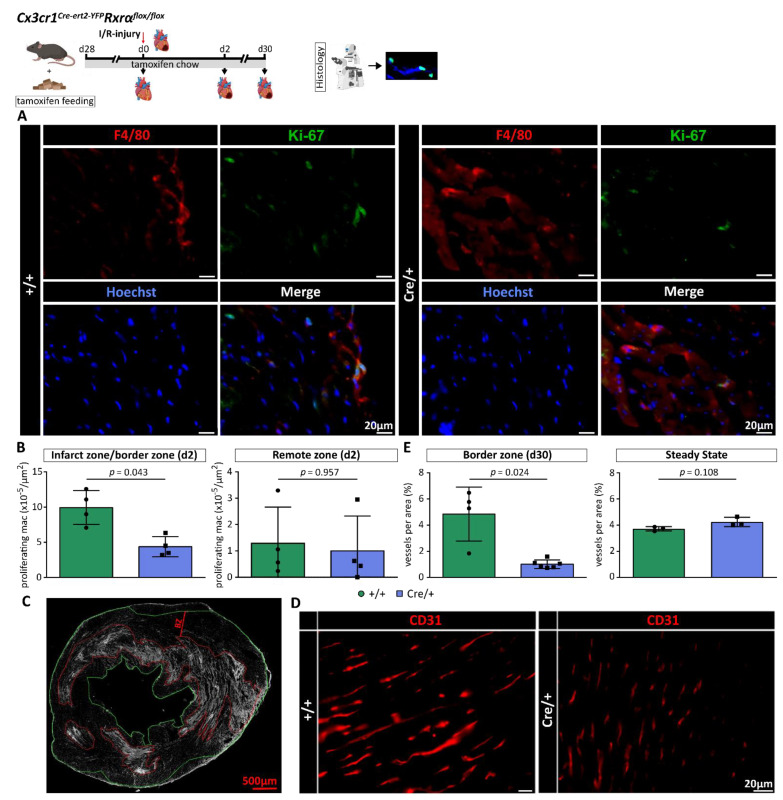
Decreased *Rxrα* expression is linked to a reduction in macrophage proliferation and vessel density in the heart after I/R injury. (**A**) IF staining to detect proliferating macrophages (F4/80: macrophage marker, Ki-67: proliferation marker, Hoechst: nuclear counterstain); (**B**) comparison of proliferating cardiac macrophages on d2 after MI between *Cx3cr1ΔRxrα* and control mice; (**C**) wheat Germ Agglutinin staining to detect the IZ using a WGA-Alexa Fluor™ 350 conjugated antibody; (**D**) visualization of cardiac vessel density using a CD31 antibody; (**E**) quantification of cardiac vessel density in the border zone on d30 after MI in *Cx3cr1ΔRxrα* compared to control mice. Flowchart was created with BioRender.com. BZ: border zone; control (+/+): control mice [littermates with a floxed Rxrα allele but without Cre expression]; *Cx3cr1ΔRxrα* (Cre/+): mice with a floxed Rxrα allele and Cre expression; IF: immunofluorescence microscopy; I/R injury: Ischemia/Reperfusion injury; IZ: infarct zone; mac: macrophages; MI: myocardial infarction; Rxr: Retinoid X receptor; WGA: Wheat Germ Agglutinin.

In order to study the effect of a myeloid-specific reduction in *Rxrα* expression on proliferation of cardiac macrophages, we performed histological analysis of cardiac tissue on d2 after MI (Methods, Figure 5A). Proliferation of cardiac macrophages was assessed in different areas of the heart, namely the infarct zone (IZ)/border zone (BZ) and remote zone (RZ). The number of proliferating macrophages in the IZ/BZ of *Cx3cr1ΔRxrα* animals was significantly lower than in the control mice. We could not detect any differences between both groups in the RZ (Figure 5B).

Furthermore, we examined whether a decreased number of proliferating cardiac macrophages is associated with a reduction in total cardiac macrophage counts. FACS analysis, performed on d1 after MI, did not reveal differences in macrophage numbers per mg cardiac tissue between *Cx3cr1ΔRxrα* and control mice (Figure S1).

Overall, a reduced expression of *Rxrα* in myeloid cells is associated with lower numbers of proliferating macrophages in the infarcted heart early after ischemic injury.

### 3.7. RXRα Is Involved in Regulation of Angiogenesis after MI

Macrophages contribute to angiogenesis by releasing pro-angiogenic mediators [6]. Previous studies indicate that the RXR in macrophages seems to be involved in regulation of angiogenesis [14].

We therefore analyzed the density of cardiac blood vessels in the BZ (defined as the area 500 µm around the IZ (Figure 5C,D) on d30 after MI. In this regard, the area of vessels was measured and divided by the total area. Quantification of vessel density in the BZ revealed a significantly lower proportion of vessels in *Cx3cr1ΔRxrα* compared to the control animals. No relevant differences in cardiac vessel density between *Cx3cr1ΔRxrα* and control mice could be detected during baseline conditions (Figure 5E).

In summary, decreased *Rxrα* expression in monocytes and macrophages leads to a reduced vascularization in the BZ after MI.

### 3.8. Reduced Rxrα Expression in Myeloid Cells Has No Impact on the Cytokine and Chemokine Profile in the Infarcted Heart on d1 after MI

**Figure 6:**
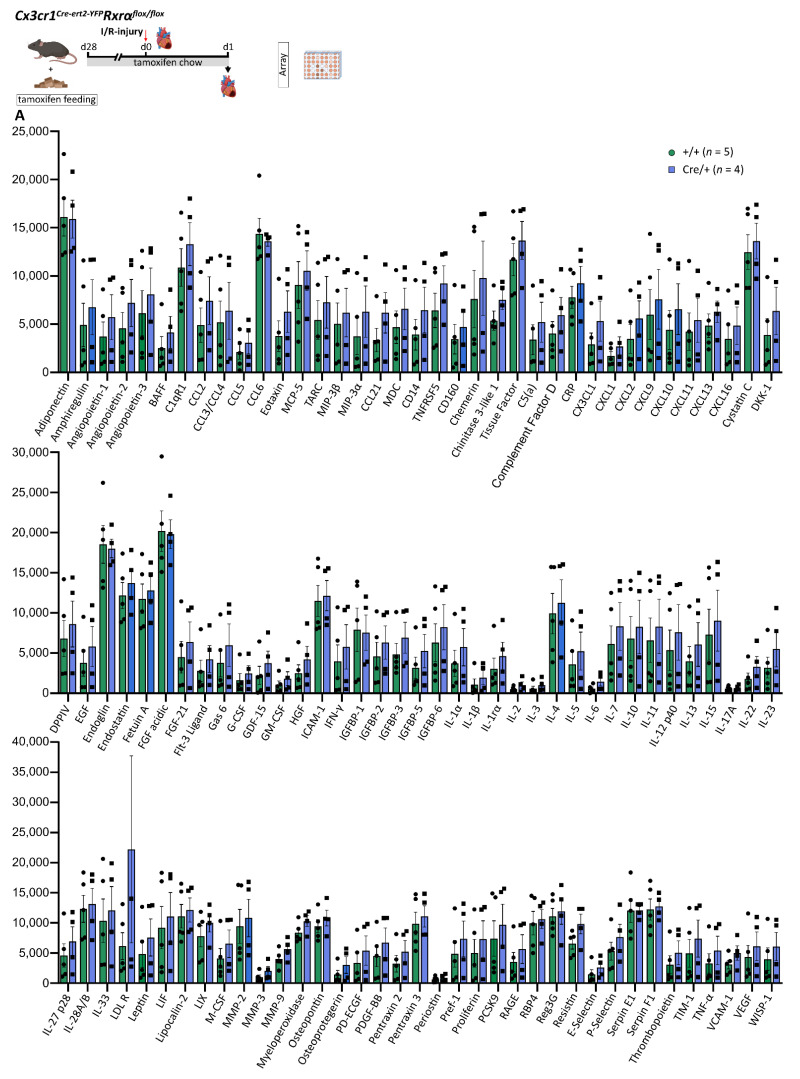
**A** Reduced expression of Rxrα in myeloid cells has no impact on cardiac cytokine and chemokine profiles on d1 after I/R injury. Cardiac cytokine and chemokine concentrations assessed by signal intensities using the Proteome Profiler^TM^ Mouse XL Cytokine Array Kit. Analytes are summarized in supplementary Table S2. Flowchart was created with BioRender.com. control (+/+): control mice [littermates with a floxed Rxrα allele but without Cre expression]; *Cx3cr1ΔRxrα* (Cre/+): mice with a floxed Rxrα allele and Cre expression; I/R injury: Ischemia/Reperfusion injury; Rxr: Retinoid X receptor.

RXRs are known to contribute to the regulation of chemokine and cytokine expression in the course of inflammation [10,11]. Anti-inflammatory effects of RXRs could be detected, making RXRs a promising target for immunomodulatory therapies [12,13,15]. Using a Proteome Profiler^TM^ Mouse XL Cytokine Array (Methods) we performed broad characterization of cardiac cytokine- and chemokine profiles in the infarcted area on d1 after MI and compared the expression in the ischemic tissue of *Cx3cr1ΔRxrα* and control mice. Comparison of 110 cytokines, chemokines, and growth factors did not yield significant differences between the two groups (Figure 6).

Thus, based on our studies, reduction of *Rxrα* expression in myeloid cells does not seem to have a substantial impact on cytokine and chemokine levels in the cardiac tissue after acute MI.

## 4. Discussion

Advances in treatment of ischemic heart disease over recent years have improved outcomes and reduced mortality after MI. However, a considerable number of patients develop heart failure after an ischemic event, and ischemic heart disease is still a leading cause of death worldwide [1,32]. The immune system is involved in cardiac remodeling after MI, and myeloid cells play an important role in the course of acute inflammation after MI [2,27]. Tight regulation of the immune response is crucial as immune dysregulation has a negative impact on outcomes after an ischemic event [2,3]. RXRs, as part of the nuclear receptor family, are expressed in different tissues and are involved in immune regulation [9,12,13,17,33]. Thus, RXRs might be promising targets for immunomodulatory therapies. Previous studies assessed the function of RXRs in health and disease; however, the impact of a reduced *Rxrα* expression in myeloid cells on cardiac remodeling after MI has not been investigated to date [7,8,9,10,11,12,14,18,29].

By specifically reducing *Rxrα* expression in myeloid cells with a tamoxifen-induced Cre-lox-system, we were able to show that a reduction of RXRα levels in monocytes and macrophages negatively impacts cardiac remodeling after myocardial infarction. Lower expression of *Rxrα* promotes apoptotic cell accumulation in the myocardium, reduces cardiac macrophage proliferation and angiogenesis in the infarct border zone/infarct area, impacts monocyte/macrophage subset composition, and is associated with a higher total perfusion deficit on day 30 after MI.

Positive implications of RXR agonists on outcomes after ischemic injuries have been previously described. Application of bexarotene after ischemic stroke decreased the infarct size and was associated with improved post-stroke recovery [18,29]. Accordingly, our result from FDG-PET analysis revealed a significantly greater infarct region on d30 after MI in *Cx3cr1ΔRxrα* in comparison to control mice, pointing towards a relevant function of RXRα in myeloid cells during remodeling processes after an acute ischemic injury.

Angiogenesis is crucial for physiological remodeling after myocardial infarction [34]. Especially, vessel formation in the BZ is an elementary process to provide sufficient oxygen to vital myocardium, reducing the expansion of the infarct core [35]. Macrophages promote cardiac angiogenesis by releasing angiogenic molecules (e.g., VEGF-A). Depletion of cardiac macrophages significantly reduces cardiac vessel formation, which leads to worse outcomes after myocardial injury [6]. Previous studies have analyzed the role of RXRs in angiogenesis. Treatment of macrophages with RXR agonists could induce vessel formation by upregulation of proangiogenic molecules such as VEGF-A [14]. In line with that, we detected a decreased vessel density in the BZ of *Cx3cr1ΔRxrα* mice on d30 after MI in relation to controls. These findings are consistent with former studies reporting an increase in proangiogenic molecules and in vessel formation in the infarct area, whereas expression of VEGF-A in the non-infarcted myocardium remained unchanged [36]. In contrast to Daniel et al. [14], we could not detect any changes in cardiac VEGF-A concentrations on d1 after MI. However, differences in VEGF-A expression at different time points could still account for the lower vessel density in the infarct border zone on d30 after MI. Reduced vessel density might be one factor contributing to an increase in the infarct size on d30 in *Cx3cr1ΔRxrα* compared to control mice.

Phagocytosis and apoptosis are important components of a regulated immune response. Uptake of cell debris by macrophages triggers an anti-inflammatory response and maintains self-tolerance [17,37,38]. Roszer et al. identified an impaired phagocytic activity of macrophages as one crucial factor contributing to a dysregulated immune response and loss of self-tolerance in *Rxrα*-KO mice in kidney disease. Impaired phagocytosis leading to a reduced cell uptake was associated with a higher number of apoptotic cells in the kidney [17]. In line with this, the phagocytic function of monocytes was reduced in *Cx3cr1ΔRxrα* mice, and we could detect higher numbers of apoptotic cells in the infarct area of *Cx3cr1ΔRxrα* compared to the control mice on d1 after MI as an indicator of immune dysregulation after MI associated with an increased infarct size.

Cardiac macrophages have the ability to proliferate and self-maintain locally [5]. While the antiproliferative effect of RXR agonists is already used in leukemia treatment, the role of RXRα in myeloid cells regarding their local proliferation remains insufficiently understood [30,31]. However, previous studies indicate that treatment of macrophages with retinoic acid leads to an upregulation of anti-apoptotic molecules and a reduced expression of pro-apoptotic signals [8]. Our data revealed that the number of proliferating macrophages is reduced in the infarct and border zone of *Cx3cr1ΔRxrα* in relation to control mice during the acute state after MI. Cardiac macrophages are an important part of the physiological immune response after cardiac injury, which is emphasized by the fact that depletion of cardiac macrophages leads to worse outcomes [6]. Even though we could find reduced proliferation of cardiac macrophages in *Cx3cr1ΔRxrα* mice, no changes in overall cardiac macrophage count could be detected. As we did not differentiate between resident cardiac macrophages and monocyte-derived macrophages, it is conceivable that a reduction in proliferating resident macrophages is associated with increased infiltration of monocyte derived macrophages, which would be in line with the reduced proportion of Ly6c^hi^ monocytes in the blood of *Cx3cr1ΔRxrα* mice. Based on previous studies, Ly6c^hi^ monocytes can contribute to different cardiac macrophage subsets and a regulated biphasic monocyte, and macrophage immune response after MI is crucial for adequate cardiac remodeling [2,3,27,39]. It remains to be determined whether a reduced *Rxrα* expression has an effect on the proportion of resident and monocyte-derived macrophages after MI, leading to a dysregulated immune response contributing to an enlargement of the infarct area.

Moreover, FACS analysis revealed changes in cardiac macrophage populations in mice expressing reduced levels of RXRα. Increased proportions of MHCII^hi^ macrophages could be detected in *Cx3cr1ΔRxrα* animals after MI. The MHCII complex is involved in antigen presentation and immune cell activation during acute inflammation; thus, differences in MHCII expression could have an impact on the course of the acute immune response after MI [3]. Beyond that, the higher amount of MHCII^hi^Ly6c^lo^ macrophages in the heart of *Cx3cr1ΔRxrα* compared to control mice could indicate increased monocyte-to-macrophage differentiation due to excessive inflammation. Taken together, future studies will be necessary to investigate the effect of the shift in monocyte and macrophages subpopulations on acute inflammation and on outcomes after MI.

The main limitation of our study is that we only achieved a reduction of *Rxrα* expression in myeloid cells by around 50%. Moreover, the missing differentiation between resident cardiac macrophages and bone marrow-derived macrophages, as well as between classical and non-classical monocytes, are other drawbacks. However, we show for the first time that reduced *Rxrα* expression in myeloid cells impacts cardiac remodeling after myocardial infarction and could identify an association with immune dysregulation leading to an increase in infarct size. Overall, further investigations with a more efficient reduction in *Rxrα* expression and future studies assessing the benefit of RXRα agonists in the course of acute MI will be necessary.

## 5. Conclusions

In summary, RXRα in myeloid cells is involved in immune regulation in the course of acute inflammation after MI by modulation of phagocytosis, cell apoptosis, angiogenesis, macrophage proliferation, and monocyte/macrophage subset composition. Reduction of *Rxrα* expression in monocytes and macrophages negatively impacts cardiac remodeling after myocardial infarction as indicated by an increase in infarct size. Thus, RXRα might be a promising target for regulating the immune response after MI in order to improve outcomes.

## Data Availability

Further information and requests for resources and data should be directed to and will be fulfilled by the lead contacts Christian Schulz (christian.schulz@med.uni-muenchen.de) and Tobias Weinberger (tobias.weinberger@med.uni-muenchen.de).

## Supplementary Materials

The following supporting information can be downloaded at: https://www.mdpi.com/article/10.3390/biomedicines10061274/s1, Figure S1: Additional analysis of monocyte and macrophage subpopulations; Table S1: Antibodies and dyes used for FACS (sorting) and immunofluorescence microscopy; Table S2: Cytokines, chemokines, and growth factors assessed by the Proteome ProfilerTM Mouse XL Cytokine Array Kit.
