# Supplementary material for: Modulation of Rxrα Expression in Mononuclear Phagocytes Impacts on Cardiac Remodeling after Ischemia-Reperfusion Injury
Source: Biomedicines. 2022 May 30;10(6):1274. doi: 10.3390/biomedicines10061274 (PMC9219801; doi:10.3390/biomedicines10061274)
Supplement: Supplementary file 1 [file biomedicines-10-01274-s001.zip › biomedicines-1720800-supplementary.pdf]

Figure S1

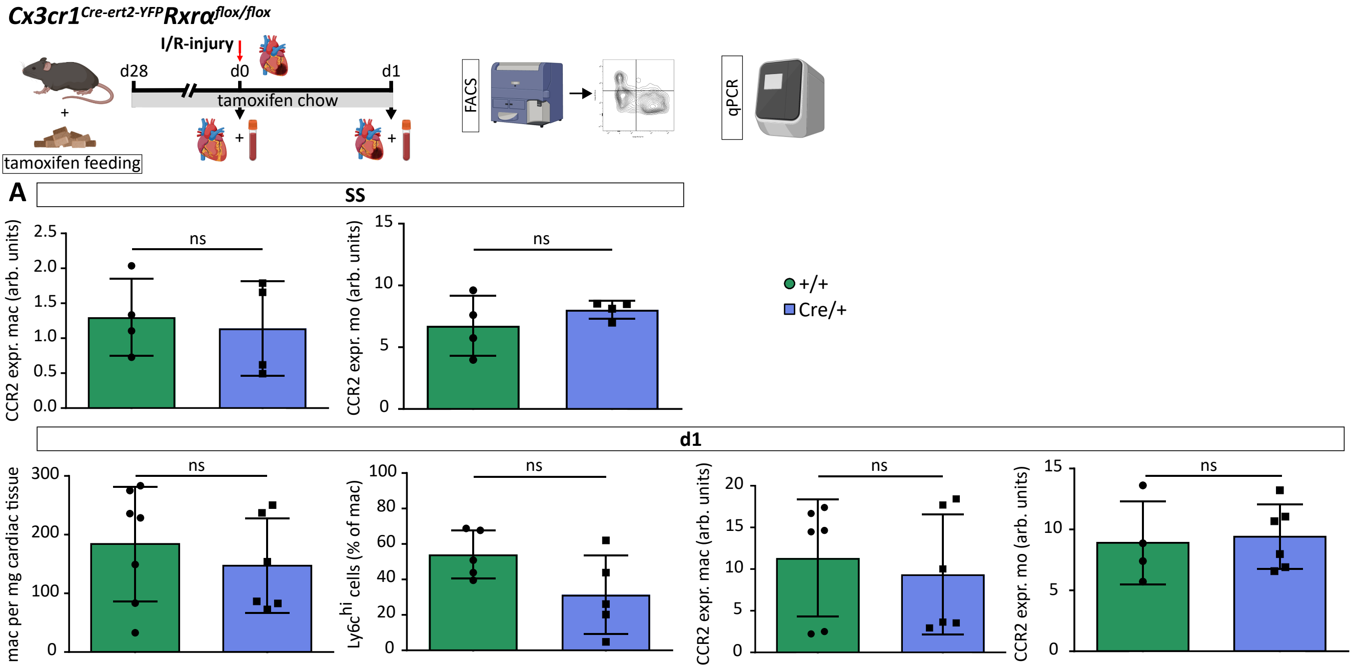
Cx3cr1Cre-ert2-YFP Rxrαflox/flox
I/R-injury
d28
d0
d1
tamoxifen chow
+
tamoxifen feeding
FACS
qPCR
A
SS
CCR2 expr. mac (arb. units)
ns
CCR2 expr. mo (arb. units)
+/+
Cre/+
d1
mac per mg cardiac tissue
Ly6chi cells (% of mac)
CCR2 expr. mac (arb. units)
CCR2 expr. mo (arb. units)

Table S1

Antibody/Dye	Company	Ref. no.	Dilution
**FACS - Heart/brain**			
PerCP/Cyanine5.5 anti-mouse CD45 antibody	BioLegend	103132	1:100
PE anti-mouse NK-1.1 antibody	BioLegend	108708	1:100
PE anti-mouse Ly-6G Antibody	BioLegend	127608	1:100
PE anti-mouse TCR β chain Antibody	BioLegend	109208	1:100
PE anti-mouse TER-119/Erythroid Cells antibody	BioLegend	116208	1:100
PE anti-mouse CD11c antibody	BioLegend	117308	1:100
APC anti-mouse CD64 (FcγRI) antibody	BioLegend	139306	1:100
Brilliant Violet 421™ anti-mouse F4/80 antibody	BioLegend	123132	1:50
**Subpopulations**			
PE/Cy7 anti-mouse I-A/I-E antibody	BioLegend	107630	1:100
Brilliant Violet 510™ anti-mouse Ly-6C antibody	BioLegend	128033	1:100
**FACS - Blood**			
APC anti-mouse CD115 (CSF-1R) antibody	BioLegend	135510	1:100
**Subpopulations**			
Brilliant Violet 421™ anti-mouse Ly-6C antibody	BioLegend	128032	1:100
**IF - Proliferation of cardiac macrophages (d2)**			
Unconjugated F4/80 antibody	ThermoFisher	14-4801-82	1:50
Cy™3 AffiniPure Goat Anti-Rat IgG (H+L)	Jackson ImmunoResearch	112-165-003	1:200
Ki67/MKI67 antibody	Novus Biologicals	NB500-170	1:50
Goat anti-Rabbit IgG (H+L) Cross-Adsorbed Secondary antibody Alexa Fluor 488	ThermoFisher	A-11008	1:200
Hoechst 33342	ThermoFisher	H3570	1:1000
**IF - Cardiac neovascularization (d30)**			
Rat Anti-Mouse CD31 antibody	BDBioscience	550274	1:25
Cy™3 AffiniPure Goat Anti-Rat IgG (H+L)	Jackson ImmunoResearch	112-165-003	1:200
Wheat Germ Agglutinin Alexa Fluor™ 350 conjugated antibody	ThermoFisher Scientific	W11263	1:100

Table S2

Analyte	Gene ID	Alternate nomenclature
Adiponectin/Acrp30	11450	AdipoQ
Amphiregulin	11839	AR, SDGF
Angiopoietin-1	11600	Ang-1, Angpt1
Angiopoietin-2	11601	Ang-2, Angpt2
Angiopoietin-like 3	30924	ANGPT-L3
BAFF/BLyS/TNFSF13B	24099	CD257, TALL1, THANK, ZTNF4
C1q R1/CD93	17064	AA4 Antigen, C1q Rp, CD93
CCL2/JE/MCP-1	20296	MCAF
CCL3/CCL4/MIP-1α/β	20302/ 20303	
CCL5/RANTES	20304	SISd
CCL6/C10	20305	MRP-1
CCL11/Eotaxin	20292	
CCL12/MCP-5	20293	
CCL17/TARC	20295	
CCL19/MIP-3β	24047	ELC
CCL20/MIP-3α	20297	exodus-1, LARC
CCL21/6Ckine	18829	exodus-2, SCYA21, SLC, TCA-4
CCL22/MDC	20299	ABCD-1, MDC, STCP-1
CD14	12475	
CD40/TNFRSF5	21939	
CD160	54215	Natural killer cell receptor BY55, NK1; NK28
Chemerin	71660	RARRES2, TIG-2
Chitinase 3-like 1	12654	CHI3L1, Cgp39, YKL40
Coagulation Factor III/Tissue Factor	14066	TF, CD142, Thromboplastin
Complement Component C5/C5a	15139	C5/C5a
Complement Factor D	11537	Adipsin, C3 convertase activator, Properdin factor D
C-Reactive Protein/CRP	12944	
CX3CL1/Fractalkine	20312	FKN, Neurotactin
CXCL1/KC	14825	CINC-1; GRO alpha; KC; MGSA-alpha
CXCL2/MIP-2	20310	GROβ, GRO2, CINC-3
CXCL9/MIG	17329	CRG-10, CMK
CXCL10/IP-10	15945	CRG-2, C7
CXCL11/I-TAC	56066	H174, SCYB9B
CXCL13/BLC/BCA-1	55985	
CXCL16	66102	SRPSOX
Cystatin C	13010	ARMD11, CST3, Gamma-trace
DKK-1	13380	Dickkopf-1
DPPIV/CD26	13482	Dpp4, Dipeptidyl-peptidase IV
EGF	13645	Epidermal Growth Factor
Endoglin/CD105	13805	ENG
Endostatin	12822	Col18a1
Fetuin A/AHSG	11625	AHSG, alpha-2-HS-glycoprotein
FGF acidic	14164	FGF-1
FGF-21	56636	
Flt-3 Ligand	14256	Flt3lg
Gas 6	14456	Growth Arrest Specific
G-CSF	12985	Csf3
GDF-15	23886	MIC-1

Analyte	Gene ID	Alternate nomenclature
GM-CSF	12981	Csf2
HGF	15234	Scatter Factor, SF, Hepatopoietin-A
ICAM-1/CD54	15894	
IFN-γ	15978	IFNG
IGFBP-1	16006	
IGFBP-2	16008	
IGFBP-3	16009	
IGFBP-5	16011	
IGFBP-6	16012	
IL-1α/IL-1F1	16175	
IL-1β/IL-1F2	16176	
IL-1ra/IL-1F3	16181	IL1RN
IL-2	16183	
IL-3	16187	
IL-4	16189	B cell-stimulatory factor-1
IL-5	16191	
IL-6	16193	
IL-7	16196	
IL-10	16153	CSIF
IL-11	16156	
IL-12 p40	16160	
IL-13	16163	
IL-15	16168	
IL-17A	16171	
IL-22	50929	IL-TIF
IL-23	83430	
IL-27 p28	246779	
IL-28A/B	330496/338374	
IL-33	77125	NF HEV, DVS 27
LDL R	16835	low density lipoprotein receptor
Leptin	16846	OB
LIF	16878	
Lipocalin-2/NGAL	16819	Siderocalin, 24p3
LIX	20311	CXCL5, GCP-2, ENA-78
M-CSF	12977	CSF-1
MMP-2	17390	Gelatinase A
MMP-3	17392	Stromelysin-1
MMP-9	17395	Clg4b, Gelatinase B, GELB
Myeloperoxidase	17523	MPO
Osteopontin (OPN)	20750	Eta-1, Spp1
Osteoprotegerin/TNFRSF11B	18383	OPG, Ocif
PD-ECGF/Thymidine phosphorylase	72962	dThdPase, ECGF1, Gliostatin, MEDPS1, MNGIE
PDGF-BB	18591	
Pentraxin 2/SAP	20219	PTX2
Pentraxin 3/TSG-14	19288	PTX3
Periostin/OSF-2	50706	Fasciclin I-like, POSTN, TRIF52
Pref-1/DLK-1/FA1	13386	DLK1, pG2, ZOG
Proliferin	18811	MRP
Proprotein Convertase 9/PCSK9	100102	NARC-1
RAGE	11596	AGER
RBP4	19662	Retinol-Binding Protein 4

Analyte	Gene ID	Alternate nomenclature
Reg3G	19695	PAP3
Resistin	57264	ADSF, FIZZ3
E-Selectin/CD62E	20339	ELAM1, LECAM2, Sele
P-Selectin/CD62P	20344	GMP-140, LECAM3, Selep
Serpin E1/PAI-1	18787	Nexin, PLANH1
Serpin F1/PEDF	20317	EPC-1
Thrombopoietin	21832	Tpo, MGDF
TIM-1/KIM-1/HAVCR	171283	
TNF-α	21926	TNFSF1A
VCAM-1/CD106	22329	
VEGF	22339	VEGF-A, VPF
WISP-1/CCN4	22402	
